# 3D imaging of proximal caries in posterior teeth using optical coherence tomography

**DOI:** 10.1038/s41598-020-72838-2

**Published:** 2020-09-25

**Authors:** Yasushi Shimada, Michael F. Burrow, Kazuyuki Araki, Yuan Zhou, Keiichi Hosaka, Alireza Sadr, Masahiro Yoshiyama, Takashi Miyazaki, Yasunori Sumi, Junji Tagami

**Affiliations:** 1 Department of Operative Dentistry, Graduate School of Medicine, Dentistry and Pharmaceutical Sciences, Okayama University, 2-5-1 Shikata-cho, Kita-ku, Okayama, 700-8525 Japan; 2 Department of Cariology and Operative Dentistry, Graduate School of Medical and Dental Sciences, Tokyo Medical and Dental University, Tokyo, Japan; 3 Faculty of Dentistry, The University of Hong Kong, Prince Philip Dental Hospital, Hong Kong, China; 4 Department of Oral Diagnostic Sciences, Division of Radiology, Showa University School of Dentistry, Tokyo, Japan; 5 Department of Preventive Dentistry, Shanghai Ninth People’s Hospital, Shanghai Jiao Tong University School of Medicine, Shanghai, China; 6 Department of Restorative Dentistry, University of Washington School of Dentistry, Seattle, USA; 7 Department for Advanced Dental Research, Center of Advanced Medicine for Dental and Oral Diseases, National Center for Geriatrics and Gerontology, Obu, Japan; 8 Department of Conservative Dentistry, Division of Biomaterials and Engineering, Showa University School of Dentistry, Tokyo, Japan

**Keywords:** Laboratory techniques and procedures, Dental caries, Dental lasers

## Abstract

Optical coherence tomography (OCT) can create cross-sectional images of tooth without X-ray exposure. This study aimed to investigate the diagnostic accuracy of 3D imaging of OCT for proximal caries in posterior teeth. Thirty-six human molar teeth with 51 proximal surfaces visibly 6 intact, 16 slightly demineralized, and 29 distinct carious changes were mounted to take digital radiographs and 3D OCT images. The sensitivity, specificity and area under the receiver operating characteristic curve (AUC) for the diagnosis of enamel caries and dentin caries were calculated to quantify the diagnostic ability of 3D OCT in comparison with digital radiography. Diagnostic accuracy was evaluated by the agreement with histology using weighted Kappa. OCT showed significantly higher sensitivity, AUC and Kappa values than radiography. OCT can be a safer option for the diagnosis of proximal caries in posterior teeth that can be applied to the patients without X-ray exposure.

Dental caries remains a highly prevalent disease that affects more than 90 percent of adults^1^. Despite advancements in caries prevention measures, dental caries remains one of the main reasons for invasive restorative treatment of teeth^2^.

Early and accurate diagnosis of caries is of fundamental importance of “Minimal Intervention Dentistry”, because it facilitates immediate non-invasive treatment approaches to control the initiation and prevent the progression of caries^3–5^. In the early stages, the demineralization process can be reversed in the presence of bioavailable calcium, phosphate and fluoride ions in the oral environment^6^. For the surface enamel lesions, infiltration of low-viscosity resin can also be an option as a micro-invasive approach to inhibit further demineralization and to arrest lesion progress^4,7^.

On the other hand, cavitated lesions involving dentin are heavily contaminated and demineralizaed with bacteria, usually require invasive management to restore function and aesthetics^8^. Consequently, cavitation is recognized as one of the major factors for deciding whether and how to intervene the lesion^8^. Since any loss of surface integrity may provide retention sites for dental plaque, lesions with cavitation will most probably active^8,9^.

Currently, visual examination and radiographic identification are the most commonly used diagnostic tools for detecting carious lesions. The International Caries Detection and Assessment System (ICDAS), which is based on visual examination, was introduced as a novel strategy aiding diagnosis and classification of carious lesion size and subsequent treatment decisions^10^. However at posterior teeth, visual examination is hard to assess on proximal surfaces when adjacent teeth are present^8^. Visual examination is not appropriate to detect the slight cavitation of caries even under laboratory conditions^9^.

Despite the use of intraoral radiographs is a widespread aid because of the limited access for visual and tactile inspection of posterior teeth, dental radiographs exhibit low sensitivity for the detection of caries because of the superimposition of anatomical structures and artifacts^11^. Incipient proximal caries of tightly contacting surfaces produces a big challenge and results in an unacceptable proportion of false-negative results. The actual extent of carious lesions is underestimated if radiographs are solely relied on for detection^12^. Moreover, a careful optimization of radiation dose and appropriate justification for performing are required by the concept “as low as reasonably achievable” (ALARA) for dental radiographs^13^. Although the hazards caused by dental radiography are relatively small and negligible, many dentists are concerned about the safety of the radiography especially for pregnant women and infants.

Recently, near-infrared light transillumination (NILT) device was developed for the detection of initial changes in caries for posterior teeth^14–16^. NILT shows higher diagnostic performance than radiographs to detect the caries at proximal surface noninvasively in simple manner^15,16^. Since the presence of cavitation into enamel or dentin is a major factor for the decision of micro invasive or restorative treatment, imaging strategy appears necessary for the proximal surface in posterior teeth^8,9^.

Optical coherence tomography (OCT) is a noninvasive cross-sectional imaging technique that uses low-coherence interferometry to determine the echo time delay and magnitude of backscattered light reflected from a biological structure^17^. Since OCT uses near-infrared light to create cross-sectional images, it does not require any radiation doses with X-ray exposure. OCT images differentiate the optical properties of tissues, including the effects of both optical absorption and scattering^17–19^. Previous studies showed that recent OCT system has a high degree of sensitivity for detection of dental caries^20–25^, tooth cracks^26,27^, and interfacial microgaps in adhesive restorations^28–30^. Polarization sensitive OCT can measure the polarization state by light-tissue interactions^18^, and has been successfully used to image incipient caries to reduce unfavorable reflections from the tooth surface^24,25,31,32^. In contrast, reflection of light between the media having different refractive indices can effectively be utilized in intensity-based non-polarized OCT for the detection of interfacial defects for dental restorations and diagnosis of caries with cavitation^20,28,33–36^.

High-speed OCT imaging provides an increased number of cross-sectional images that are acquired in a sequence for the generation of three-dimensional (3D) volumetric data sets, which contain comprehensive structural information that enables evaluation of the feature or defect inside a structure. Currently, 3D imaging technology for OCT is being utilized in the field of dentistry and has shown excellent results with regard to the analysis of tooth cracks and marginal defects under resin composite restorations^27,37^. Several studies have demonstrated the possibility of 3D OCT for the imaging of carious lesions^38–41^. For proximal caries, OCT imaging can be applied from the occlusal surface because of the high translucency of near infrared against the enamel^42,43^. However, information of 3D imaging on the diagnostic accuracy and credibility for proximal caries is very limited. Since diagnostic accuracy relates to the ability of the test method to discriminate between the target condition and health, discriminative ability of 3D OCT for proximal caries is crucial for the decision of intervene approach. Recently, 3D images of OCT with dynamic slicing at lesion site was shown to exhibit excellent outperformance for the diagnosis of occlusal caries^44^. Despite diagnosis of caries can occur at variety of sites, the ability of diagnostic techniques to detect carious lesions on specific sites is not widely understood^12^.

Therefore, the aim of this ex vivo study was to evaluate the diagnostic accuracy of 3D imaging of intensity-based non-polarized OCT for the diagnosis of posterior proximal caries. In this study, 3D OCT images with dynamic slicing of proximal surfaces were employed. We hypothesized (H0) that 3D image of OCT would have no improvement of diagnosis for proximal caries in posterior teeth and could not provide the information for the decision of intervene approach compared with the conventional intraoral digital radiography. This null hypothesis was tested against the alternative hypothesis (H1) of difference. We first took 3D images of OCT and intraoral digital radiographs of the proximal surfaces of posterior teeth mounted in silicone blocks simulating normal anatomical position. For the OCT imaging of proximal surfaces of posterior teeth, right-angled intraoral probe designed for posterior teeth was used (Fig. 1)^42–45^. The presence and extent of proximal caries were evaluated by 13 examiners and scored using 4-rank depth scale. The sensitivity, specificity and area under the receiver operating characteristic curve (AUC) for the diagnosis of proximal caries were calculated for 3D OCT and intraoral digital radiography. Diagnostic accuracy of two diagnostic methods were compared by the agreement with histology calculated by weighted kappa. Obtained results were statistically analyzed at a significance level of α = 0.05.

**Figure 1:**
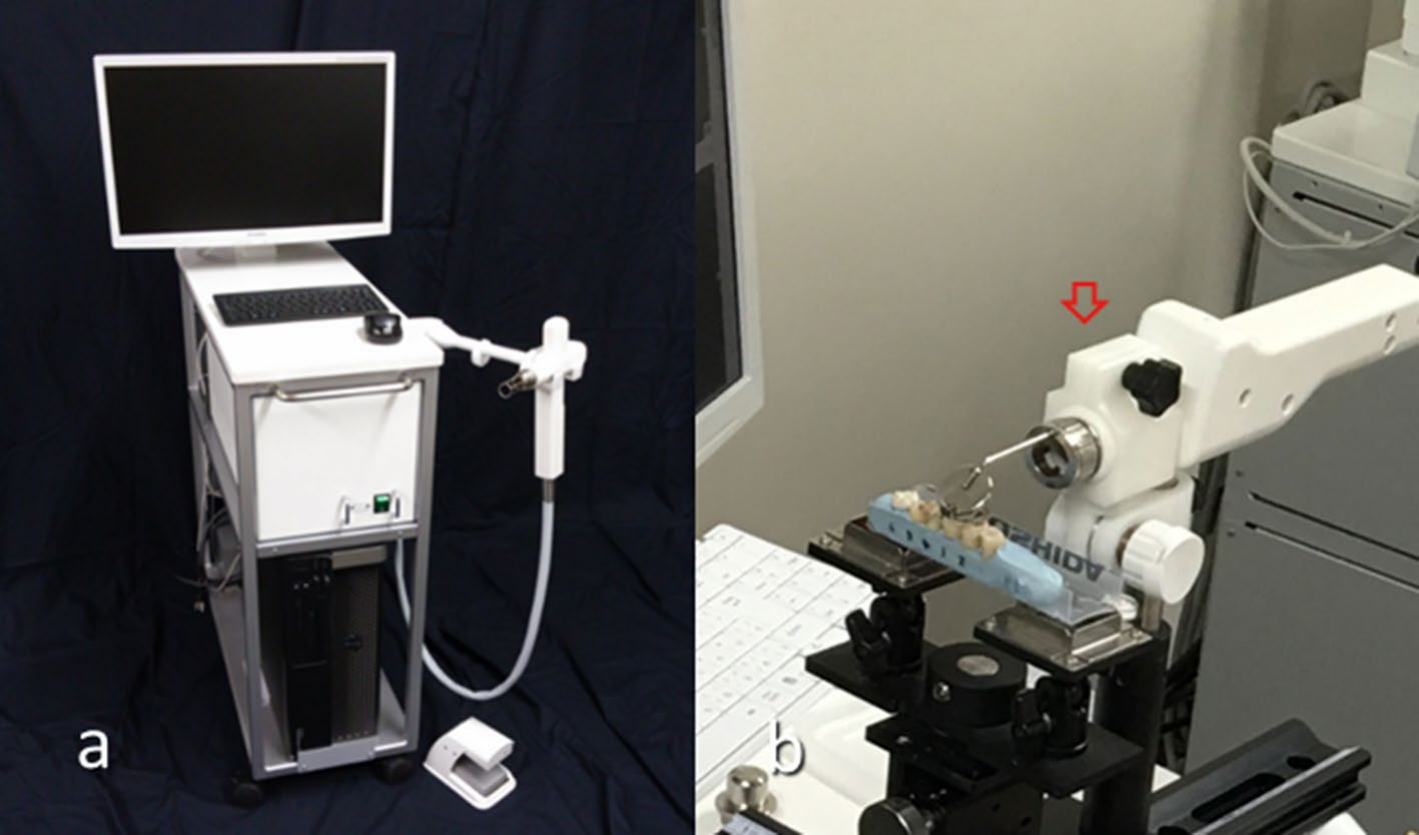
Prototype Yoshida Dental OCT (**a**) and intraoral probe with a front surface dental mirror for posterior teeth imaging (**b**, red arrow).

## Results

In this study, a total of 51 proximal surfaces of 36 molars visually with or without caries for ICDAS codes 0 to 5 were used. We validated the surfaces by the direct observation of cross-sectioned surfaces histologically at a magnification × 30. As a result, 51 surfaces included 12 intact surface (score 0), 10 enamel demineralization (score 1), 12 enamel caries (score 2), and 17 dentin caries (score 3).

**Figure 2:**
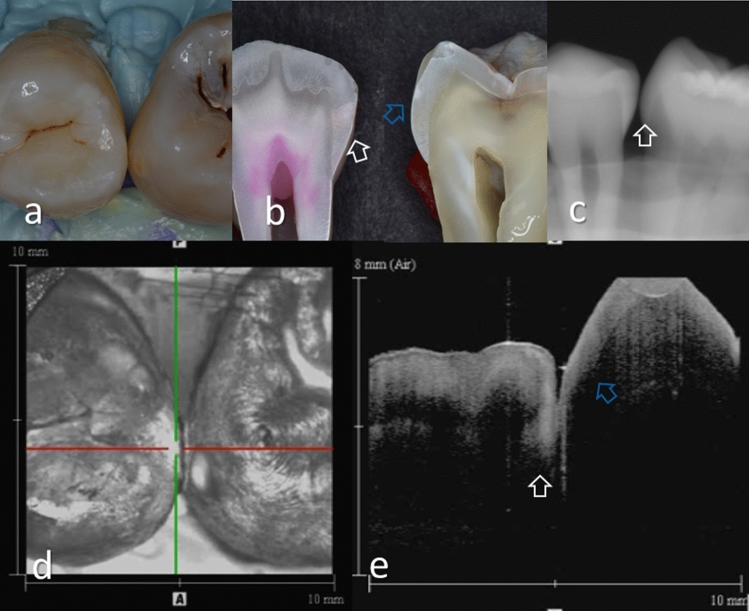
Images of teeth with enamel demineralization (score 1). (**a**) Occlusal view of proximal contacts. Histological view of the area of interest stained by 5% acid red solution. Left: Enamel demineralization penetrating deep into the enamel (white arrow, score 1). Right: Enamel demineralization localized at the superficial enamel (blue arrow, score 1). (**b**) Intraoral digital radiograph. Evidence of enamel demineralization was not found (white arrow). (**c**) En face intensity image generated by 3D OCT. (**d**) Extracted 2D image of the area along the red line in d. Left: The contour line of the proximal surface was smooth without any cavitation. The brightness at the proximal enamel was increased penetrating approximately half thickness of the enamel (white arrow). Right: The contour line of the proximal surface was smooth. The brightness of superficial enamel was slightly increased (blue arrow). The dynamic slicing 3D video is presented in Supplementary Materials: Video 1.

**Figure 3:**
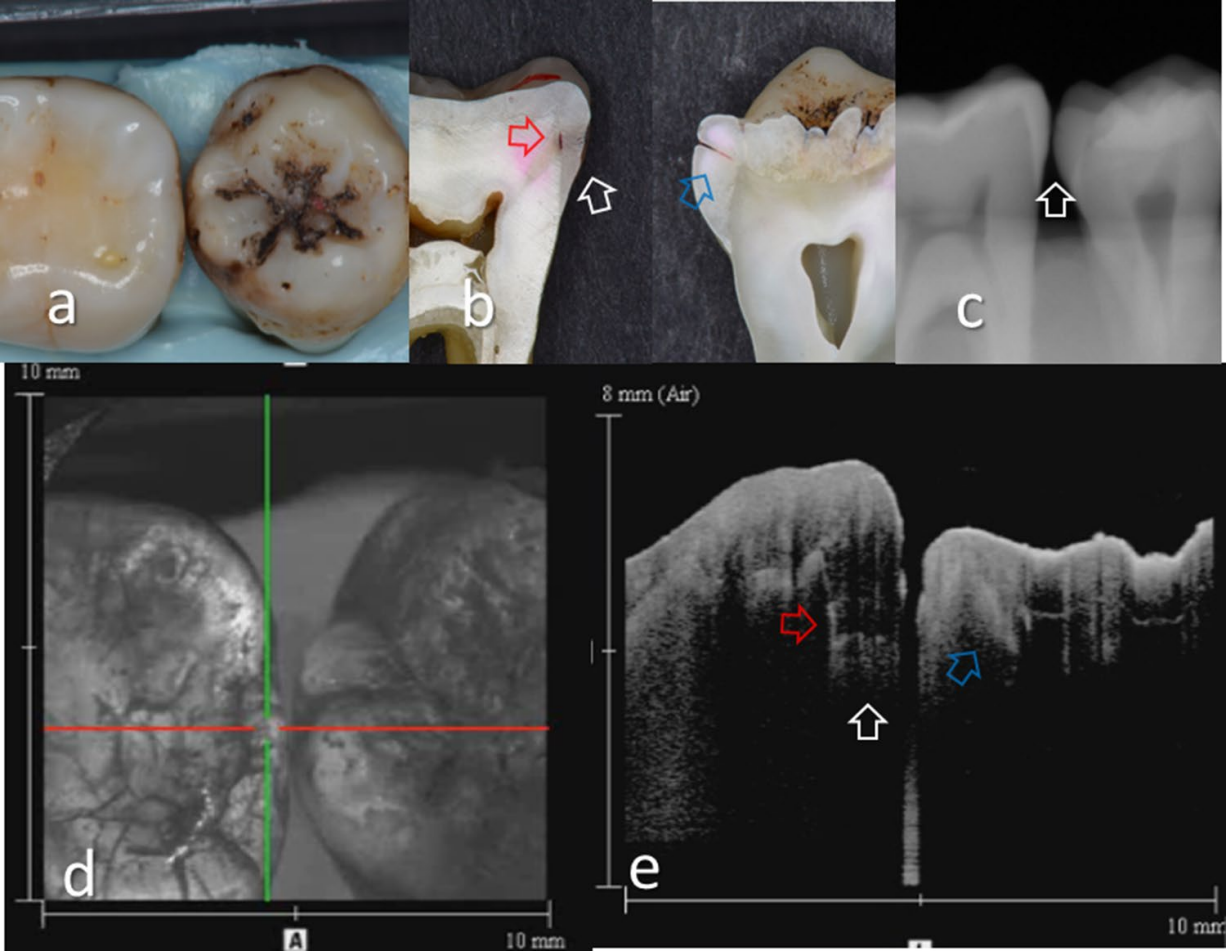
Images of teeth with enamel caries (score 2) and hidden dentin caries (score 3). (**a**) Occlusal view of proximal contacts. (**b**) Histological view of the area of interest stained by 5% acid red solution. Left: enamel caries and demineralization penetrating whole thickness of the enamel (white arrow). Incipient hidden dentin caries penetrating along DEJ (red arrow, score 3). Right: enamel caries penetrating along the developmental defects associated with surrounding enamel demineralization (blue arrow, score 2). (**c**) Intraoral digital radiograph. Evidence of caries was not clear (white arrow). (**d**) En face intensity image generated by 3D OCT. (**e**) Extracted 2D image of the area along the red line in d. Left: The brightness of proximal zone was increased penetrating the entire enamel thickness (white arrow). The brightness of the DEJ was increased because of the lesion along DEJ (red arrow). Right: The contour line of the proximal surface was slightly collapsed because of the caries. Increased light scattering due to enamel demineralization was evident (blue arrow). The dynamic slicing 3D video is presented in Supplementary Materials: Video 2.

**Figure 4:**
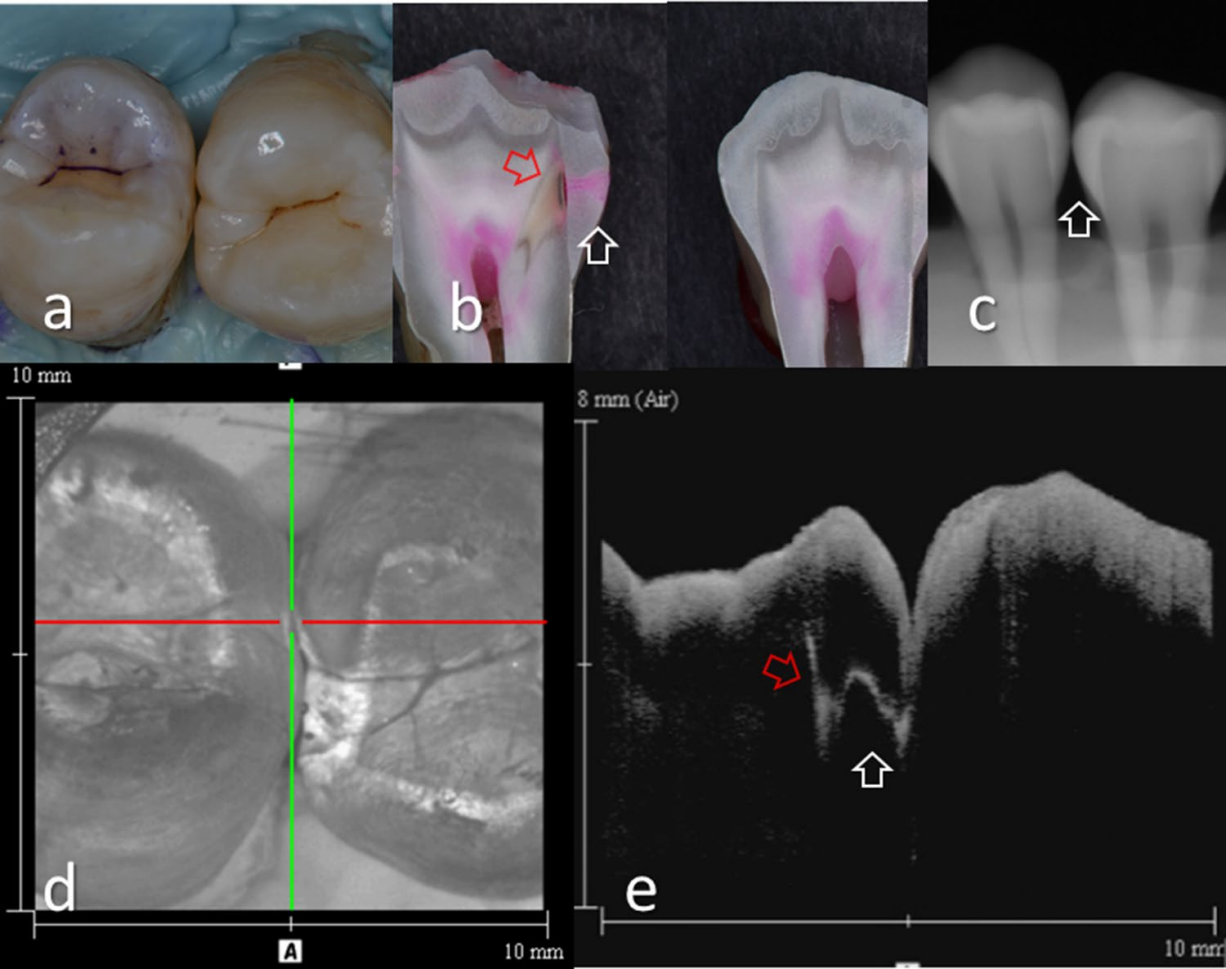
Images of teeth with intact proximal surface (score 0) and hidden dentin caries (score 3). (**a**) Occlusal view of proximal contacts. (**b**) Histological view of the area of interest stained with 5% acid red solution. Left: Enamel demineralization penetrating the entire enamel thickness. However, breakdown of enamel surface was not evident (white allow). Dentin caries with slight cavitation was observed penetrating along DEJ (red arrow, score 3). Right: Intact (score 0). (**c**) Intraoral digital radiograph. Evidence of caries was not clear (white arrow). (**d**) En face intensity image generated by 3D OCT. (**e**) Extracted 2D image of the area along the red line in d. Left: Brightness of proximal zone was significantly increased penetrating the entire enamel thickness (white arrow). The brightness of the DEJ was increased because of lateral spread of the lesion along DEJ (red arrow). Because of the slight cavitation along DEJ, intensity of brightness at DEJ appears higher than Fig. 3e left. Right: Brightness of proximal surface was not increased and almost constant. The dynamic slicing 3D video is presented in Supplementary Materials: Video 3.

**Figure 5:**
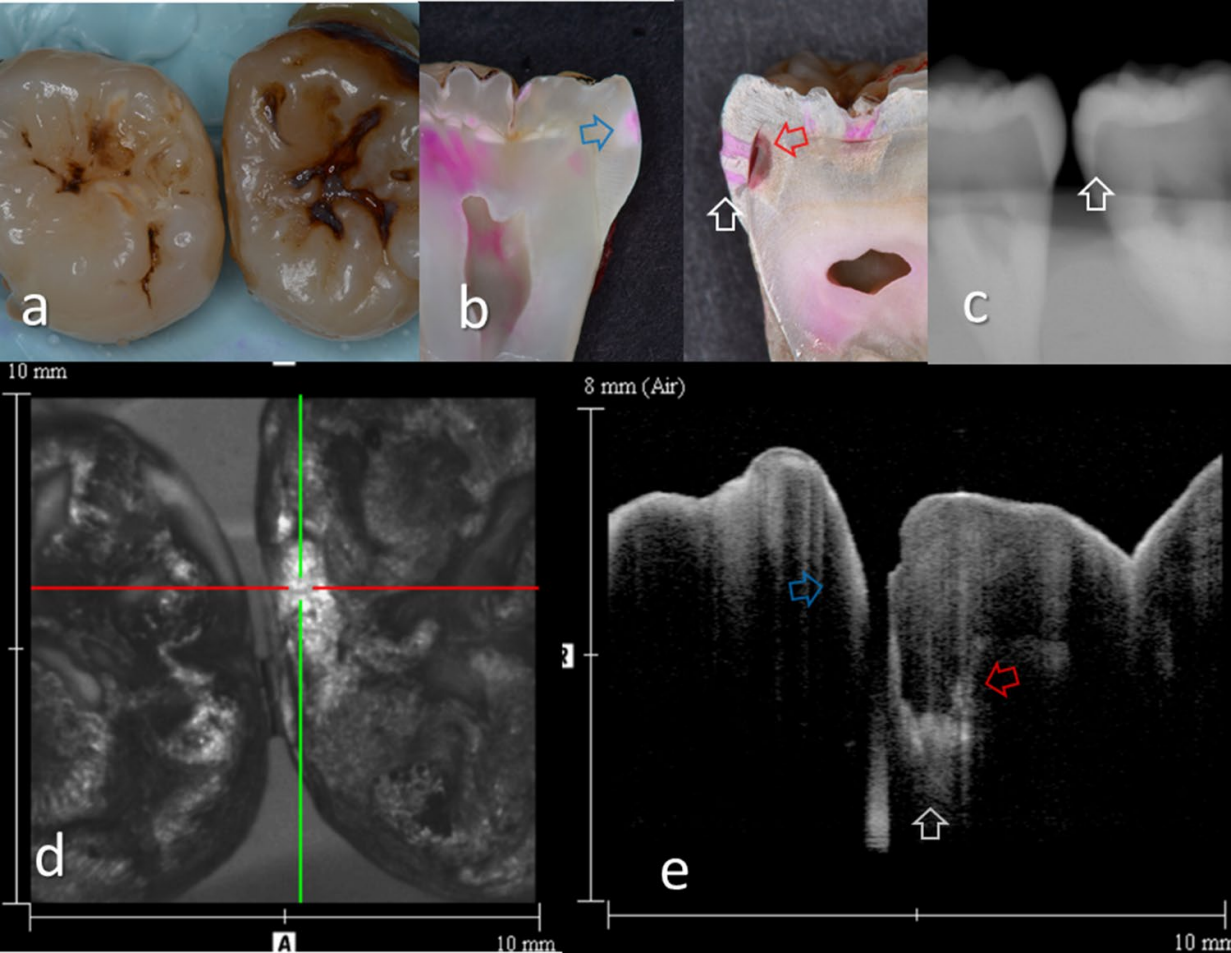
Images of teeth with enamel demineralization (score 1) and dentin caries (score 3). (**a**) Occlusal view of proximal contacts. (**b**) Histological view of the area of interest stained by 5% acid red solution. Left: Incipient enamel demineralization (blue arrow, score 1). Right: Enamel caries penetrating the entire enamel thickness (white arrow). Dentin caries with lateral spread of the lesion along the DEJ (red arrow, score 3). Cavitation was observed at DEJ. (**c**) Intraoral digital radiograph. Although the translucency of enamel was slightly increased, evidence of cavitated caries was not clear (white arrow). (**d**) En face intensity image generated by 3D OCT. (**e**) Extracted 2D image of the area along the red line in d. Left: The contour line of the proximal surface was smooth without any cavitation. The brightness of the proximal zone was increased within the enamel thickness (blue arrow). Right: The contour line of the proximal surface was slightly collapsed because of the caries. Increased light scattering due to demineralization was evident penetrating the entire enamel thickness (white arrow). The brightness of the DEJ was increased because of lateral spread of the lesion along DEJ (red arrow). The dynamic slicing 3D video is presented in Supplementary Materials: Video 4.

**Figure 6:**
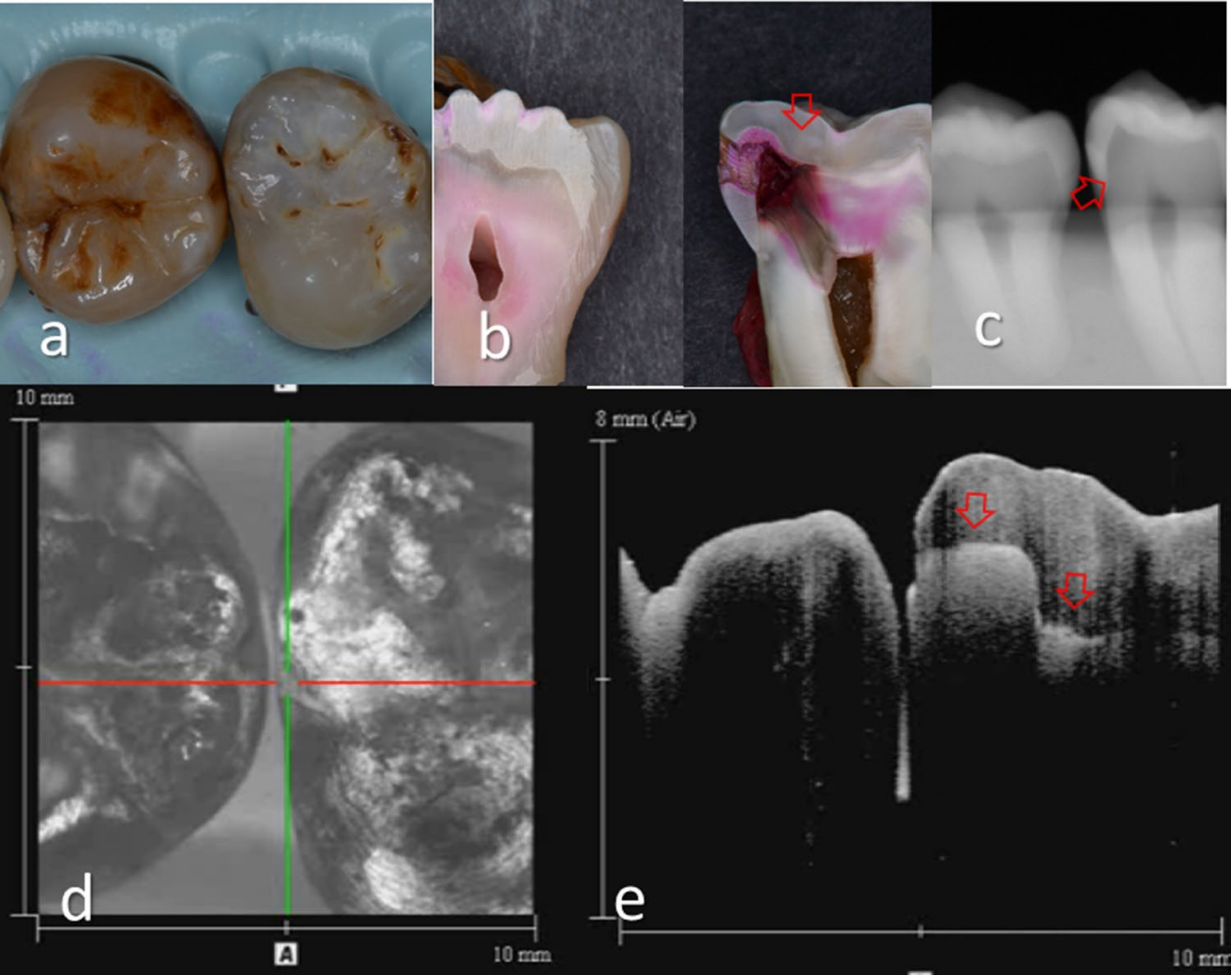
Images of teeth with intact proximal surface (score 0) and advanced dentin caries (score 3). (**a**) Occlusal view of proximal contacts. (**b**) Histological view of the area of interest stained with 5% acid red solution. Left: Intact (score 0). Right: Advanced dentin caries with distinct cavitation. (red arrow, score 3). (**c**) Intraoral digital radiograph. Dentin caries was imaged as a translucent zone penetrating approximately half thickness of the dentin (red arrow). (**d**) En face intensity image generated by 3D OCT. (**e**) Extracted 2D image of the area along the red line in b. Left: The contour line of the proximal surface was smooth without any cavitation. The brightness of the proximal zone was almost constant with no evidence of enamel demineralization. Right: There was a distinct bright line penetrating beyond DEJ (red arrows). The upper border of the hollow space due to caries showed strong light scattering. The dynamic slicing 3D video is presented in Supplementary Materials: Video 5.

**Figure 7:**
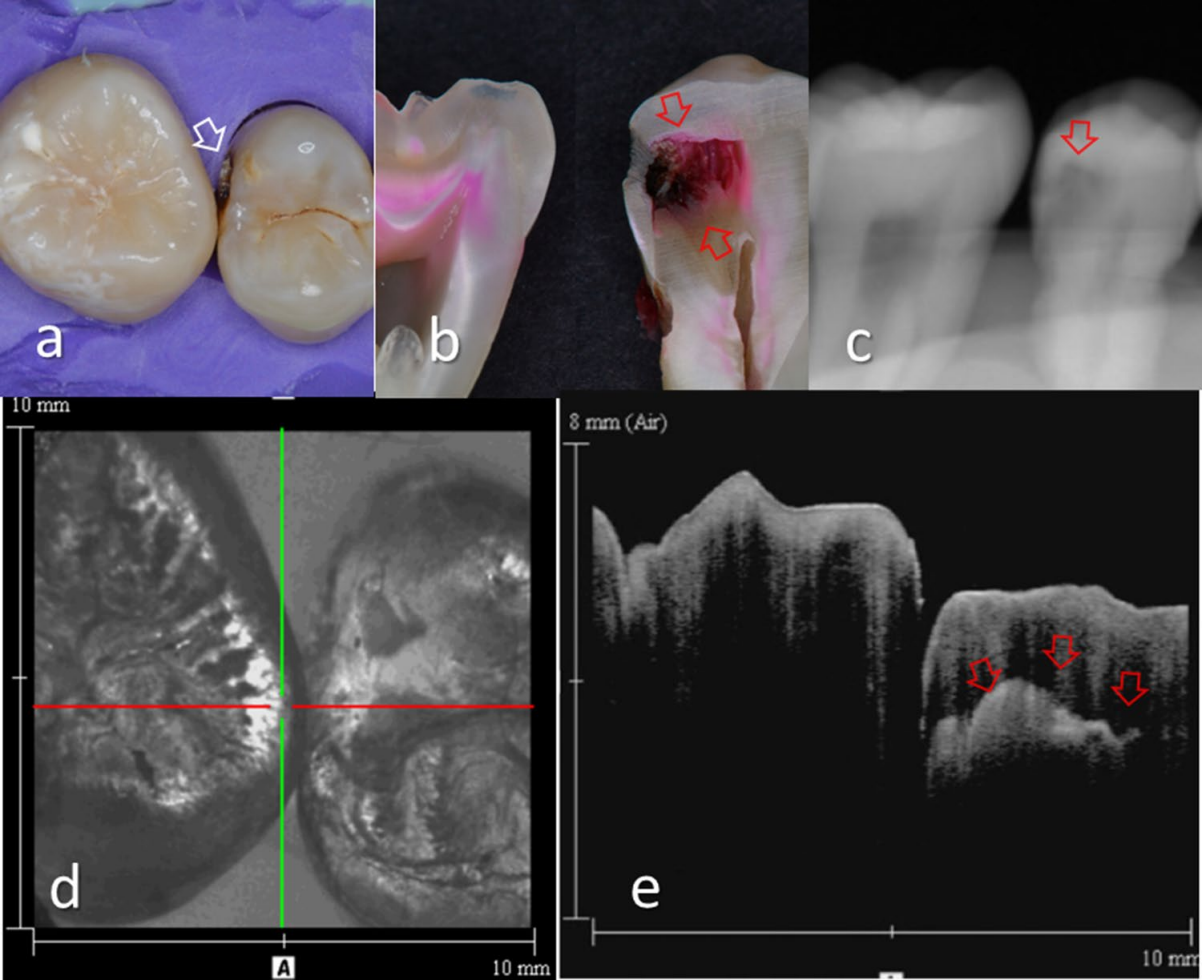
Images of teeth with intact proximal surface (score 0) and advanced dentin caries (score 3). (**a**) Occlusal view of proximal contacts. Presence of cavitation was visually accessible in this case (white arrow). (**b**) Histological view of the area of interest stained with 5% acid red solution. Left: intact (score 0). Right: Advanced dentin caries with distinct cavitation (red arrows, score 3). (**c**) Intraoral digital radiograph. Dentin caries was imaged as a translucent zone penetrating close to the pulp (red arrow). (**d**) En face intensity image generated by 3D OCT. (**e**) Extracted 2D image of the area along the red line in d. Left: The contour line of the proximal surface was smooth without any cavitation. The brightness of the proximal zone was almost constant with no evidence of enamel demineralization. Right: There was a distinct bright line penetrating beyond the DEJ (red arrows). The upper border of the hollow space due to caries showed strong light scattering. The dynamic slicing 3D video is presented in Supplementary Materials: Video 6.

Representative images of the proximal surfaces and the carious lesions are shown in Figs. 2, 3, 4, 5, 6, and 7. In OCT, the enamel surface was clearly visible, while the reflection signal from the underlying dentin was weaker and appeared as a dim image. The demineralized proximal area was easily differentiated from the sound enamel as a brighter zone with greater scattering. Representative video image of dynamic slicing from 3D OCT dataset are presented in Supplementary Materials.

The sensitivity, specificity, and AUC values of 3D OCT and intraoral digital radiography based on the validation are shown in Table 1. Agreements of 3D OCT and intraoral digital radiography with histology are shown in Table 2. 3D OCT showed higher sensitivity and AUC than intraoral digital radiography for the caries in all levels (Mann–Whitney U test, *p* < 0.05). The sensitivity of 3D OCT for enamel demineralization was 0.89, whereas for cavitated enamel caries and dentin caries were 0.87 and 0.85, respectively. For intraoral digital radiography, the sensitivity for enamel demineralization was 0.73, whereas the values for enamel caries and dentin caries were 0.59 and 0.45, respectively.

**Table 1:** Sensitivity, specificity and area under the receiver operating characteristic curve (AUC) for 3D OCT and intraoral digital radiography (IDR) for the detection of enamel demineralization, enamel caries, and dentin caries.

Examiner #	Method	Sensitivity			Specificity			AUC
		0 versus 1–3	0–1 versus 2–3	0–2 versus 3	0 versus 1–3	0–1 versus 2–3	0–2 versus 3	
1	3D OCT	0.85	0.93	0.88	0.83	0.91	0.97	0.95
	IDR	0.54	0.55	0.47	0.83	0.91	0.94	0.75
2	3D OCT	0.90	0.90	0.88	0.42	0.73	0.97	0.96
	IDR	0.59	0.52	0.29	0.83	0.77	0.88	0.69
3	3D OCT	0.92	0.97	0.82	0.83	0.82	0.97	0.95
	IDR	0.62	0.34	0.29	0.50	0.91	0.97	0.70
4	3D OCT	0.97	0.90	0.94	0.55	0.67	0.97	0.95
	IDR	0.69	0.45	0.29	0.67	0.77	0.88	0.71
5	3D OCT	0.95	0.83	1.00	0.58	0.82	0.94	0.97
	IDR	0.82	0.76	0.65	0.33	0.86	0.85	0.78
6	3D OCT	0.85	1.00	0.94	0.92	0.95	1.00	0.99
	IDR	0.82	0.76	0.47	0.58	0.95	0.94	0.78
7	3D OCT	0.87	0.90	0.88	0.83	0.91	0.97	0.97
	IDR	0.69	0.76	0.59	0.92	0.91	0.82	0.73
8	3D OCT	0.95	0.97	0.94	0.83	0.91	0.97	0.94
	IDR	0.90	0.76	0.65	0.83	0.77	0.91	0.82
9	3D OCT	0.97	0.69	0.47	0.58	0.77	0.97	0.89
	IDR	0.67	0.38	0.18	0.67	1.00	0.97	0.74
10	3D OCT	0.82	0.86	0.82	0.83	0.95	0.97	0.91
	IDR	0.74	0.59	0.41	0.50	0.91	0.97	0.72
11	3D OCT	0.90	1.00	0.88	0.75	0.95	0.97	0.96
	IDR	0.92	0.72	0.71	0.67	0.95	0.88	0.84
12	3D OCT	0.87	0.79	0.76	0.75	0.86	1.00	0.95
	IDR	0.87	0.59	0.41	0.33	0.91	0.91	0.77
13	3D OCT	0.74	0.62	0.82	0.75	0.95	0.94	0.92
	IDR	0.59	0.45	0.41	0.42	0.73	0.91	0.68
Mean (SD)	3D OCT	0.89 (0.064)	0.87 (0.11)	0.85 (0.13)	0.73 (0.14)	0.86 (0.092)	0.97 (0.016)	0.94 (0.026)
	IDR	0.73 (0.12)	0.59 (0.15)	0.45 (0.16)	0.62 (0.19)	0.87 (0.082)	0.91 (0.045)	0.75 (0.048)
*P* value, significance		*p* = 0.015, S	*p* = 0.0013, S	*p* = 0.000042, S	*p* = 0.18, NS	*p* = 0.90, NS	*p* = 0.0019, S	*p* = 0.000014, S

S: Significant difference between the values for SS-OCT and intraoral digital radiography.

NS: No significant difference between the values for SS-OCT and intraoral digital radiography.

**Table 2:** Diagnostic agreement of 3D OCT and intraoral digital radiography (IDR) results with histological findings for proximal caries (weighted kappa).

Examiner #	3D OCT	IDR
1	0.78	0.38
2	0.64	0.26
3	0.79	0.21
4	0.72	0.23
5	0.72	0.45
6	0.87	0.53
7	0.78	0.52
8	0.86	0.59
9	0.52	0.28
10	0.74	0.38
11	0.83	0.62
12	0.68	0.37
13	0.58	0.17
Mean (SD)	0.73 (0.11)	0.38 (0.15)

With regard to specificity, the values for enamel demineralization, cavitated enamel caries, and dentin caries were 0.73, 0.86, and 0.97, respectively for the 3D OCT, and 0.62, 0.87, and 0.91, respectively for intraoral digital radiography. 3D OCT showed significantly higher specificity for the detection of dentin caries than digital radiography (Mann–Whitney U-test, *p* < 0.05). For the enamel demineralization and cavitated enamel caries, no significant difference was found between the two diagnostic techniques (Mann–Whitney U test, *p* > 0.05). Considering AUC values, 3D OCT also presented higher values than intraoral digital radiography (3D OCT 0.94, intraoral digital radiography 0.75; Mann–Whitney U test, *p* < 0.05).

The agreement with histology obtained from 3D OCT and intraoral digital radiography results were 0.73 and 0.38, respectively (Table 2). 3D OCT showed higher diagnostic accuracy than digital dental radiography.

## Discussion

In this study, 3D imaging of intensity-based OCT was performed for evaluation of the proximal contact areas of molar teeth for the diagnosis of proximal caries. Null hypothesis of this study was that 3D image of OCT would have no improvement of caries diagnosis for proximal caries in posterior teeth and could not provide the image for the decision of intervene approach. Sensitivity, specificity, and AUC values for the proximal caries with different lesion depths up to dentin caries were obtained from the results by 13 dentists and compared with the results of intraoral digital radiography. Diagnostic accuracy for both methods was calculated by the agreement with histological observation. While the OCT probe used in this study was designed for the imaging of posterior teeth, distal end of OCT probe was right-angled using front surface dental mirror to scan from the occlusal aspect (Fig. 1b). On the OCT images, sound enamel appears almost transparent at the light source wavelength of approximately 1,300 nm, and the interproximal region can be displayed on the monitor from contact area or even deeper enamel.

**Figure 8:**
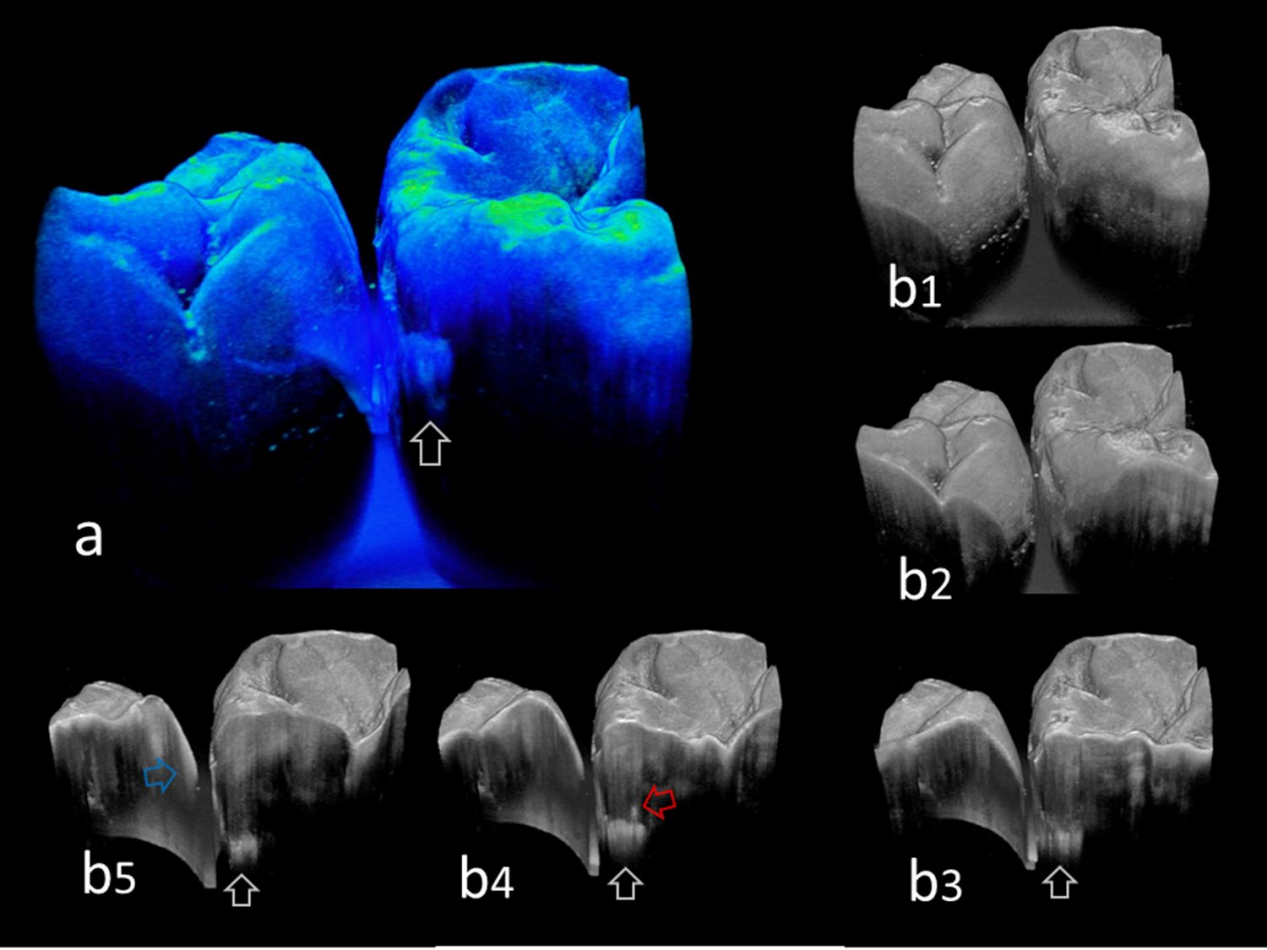
3D SS-OCT images of the same teeth in Fig. 5. (**a**) Pseudocolor image processed translucently. Presence of caries at the proximal surface was clearly imaged with the lesion location and size (white arrow). (**b1–5**) Sequential 2D images acquired in 3D view from different thickness. White arrows: enamel demineralizarion penetrating entire enamel thickness. Red arrow: lateral spread of caries along DEJ. Blue arrow: incipient enamel demineralization.

3D images generated from OCT allowed detailed examination for proximal caries by using multiplanar visualization of dynamic slicing (Figs. 2, 3, 4, 5, 6, and 7, Videos 1–6). The 3D method enabled us to observe internal tooth structures from the area of interest in several different views by changing the scanning angle and thickness (Fig. 8). Consequently, the limitation of 2D OCT imaging, such as a suboptimal projection angle that makes it difficult to differentiate a suspicious finding from an imaging artifact, can be avoided by using a variable imaging location method. As a result, 3D OCT imaging allows the clinician to recognize the tooth anatomy immediately to identify the lesion location.

Moreover, 3D OCT can image the surface topography of approximal zone, which allows the dentist to inspect the tooth surface even for the distal zone (Fig. 8). In this study, 7 proximal surfaces out of 17 dentin caries were advanced to form distinct cavitation (ICDAS code 5), in which 2 cases appears to be taken in the diagnosis easily by the suspicious looking from the digital camera image (Fig. 7). However, detecting cavitation on proximal surfaces using visual means is nearly impossible in real clinical situation when adjacent teeth are present^8^. Correctly assessing the surface integrity allows avoiding both over- and under-treatment due to false-positive and negative diagnosis^46^. Direct visual inspection examination after orthodontic elastic separation is recognized as one of the most reliable ways to detect carious cavitation, but this is not practical in most settings^8^. In 3D image of OCT, it can offer the surface topography even for the distal proximal surfaces where visual inspection is difficult to access. This power of depiction for surface topography in OCT also appears beneficial factor for the detection of cavitated lesions.

Presence of enamel caries at the proximal tooth surface was displayed as a bright zone in OCT due to the enhanced backscattering signal from the demineralized tooth structure (Figs. 2, 3). Enamel demineralization results in an increase in porosity having different refractive index to cause backscattering within the lesion body^34^. Accordingly, enamel demineralization even at the incipient stage appeared as a bright zone and could be clearly distinguished from sound enamel^21,22,45^. In addition, the depth of demineralization and caries penetration could be estimated by the location of the DEJ as a reference point on the 3D images (Figs. 2–5).

With regard to dentin caries, OCT could image the lesion as a bright zone penetrating the dentin. The penetration depth of the bright zone observed in OCT extended beyond the DEJ position, where a distinct white line along the DEJ adjacent to the caries was observed in many cases (Figs. 3–5). Whereas an intact DEJ is generally displayed as a dark line (in Figs. 4, 6, 7, Score 0 teeth) the appearance of a white line indicates involvement of the junction by the carious lesion along with structural disintegration of the enamel and dentin^43,44^. The lateral expansion of demineralization along the DEJ creates microgaps or defects where strong reflection of light occurs to show the DEJ as a distinct white line in intensity-based OCT (Figs. 4, 5). This image obtained from intensity-based OCT appears similar phenomenon to the image of microgaps formed under the dental restorations^28,35,36^. Because the Fresnel reflections from the gaps under the dental restorations, intensity-based OCT could detect the microgaps of only a few micrometers in height, which is below the optical resolution of image pixel for OCT^28,35^. When the caries progresses and results in cavitation, the signal intensity at the margin of the defect is also enhanced resulting in the appearance of a bright cavity border that allows easy differentiation from a demineralized lesion without cavitation (Figs. 6, 7). In 3D OCT, since the lesion location and size were clearly demonstrated as a 3D image, the examiner could easily recognize the strong reflection from the DEJ and the border of the cavitated lesion displayed as a distinct white line. Consequently, false positive findings could be reduced and excellent results in terms of both sensitivity and specificity were obtained especially for the detection of cavitated enamel and dentin caries. Furthermore, the unique ability of OCT imaging to detect microstructual defects in the tooth enables to depict the hidden lesion occurred at the proximal subsurface^43,44^. Figure 4 showed visibly enamel demineralization with ICDAS code 3, but exhibited a clear separation at DEJ, signifying the lateral spread of the bacteria at DEJ and deeper penetration of the lesion that seemed to be a shallow cavitation when being observed histologically.

In this laboratory study, we used extracted teeth to assess the diagnostic accuracy of 3D OCT for proximal caries. Six of 13 examiners had experience in OCT imaging and 7 examiners were the first-time viewers. The obtained results after the 1-h session for OCT imaging showed no significant difference for the values of diagnostic accuracy (sensitivity, specificity, AUC, kappa) between the experienced and first-time groups, (Mann–Whitney U-test, *p* > 0.05). The mean sensitivity over the participating examiners of 3D OCT for the detection of dentin caries (Table 1, score 1–2 vs. 3) was 0.85 (± 0.13), a dramatic improvement over the value for 2D OCT in previous in vivo study (Sensitivity for dentin caries: 0.56)^43^. Even though the test method discrepancy exists between ex vivo and in vivo, the superiority of 3D OCT for the detection of dentin caries even maintaining high specificity appears due to the enhanced interpretation of lesion size and depth on the 3D image.

Moreover, the mathematical algorithm for 3D imaging installed in the OCT device can improve visualization of cavitated lesion boundaries or the DEJ. A raw 3D image is generated as a 3D array of voxels or pixels with a grayscale range from 0 to 65,535 in a 16-bit pixel case. 3D imaging requires defined object boundaries especially for the creation of 3D surface models by signal filtering. In the OCT system used in the current study, the noise is smoothed by using a Gaussian function on the image pixels, and the edge intensity and edge direction are extracted by an edge extracting filter. The image segmentation of pixel detection mathematically appears to minimize the artifacts such as speckle-noise, whereas images obtained by coherent imaging systems are inherently corrupted.

The superiority of 3D imaging over 2D imaging for the diagnosis of medical diseases has been reported in many fields. In ophthalmology, 3D assessment of OCT can successfully image deeper or outer retinal layers and diagnose widespread retinal damage^47,48^. There are several lines of evidence supporting the improvement of caries detection using 3D imaging in radiography. The rate of detection of proximal caries using standard 2D bitewing radiographs is about 60%^12^. On the other hand, 3D cone-beam computed tomography (CBCT) affords better results for cavitated proximal lesions with an approximately 30% increase in detection rate^49^. However, CBCT is not considered suitable for use in routine clinical practice because of the high effective radiation doses of 11–77 μSv, depending on the field of view, exposure parameters, and region examined^50^. OCT does not require any radiation exposure to the patient, it is a safe option avoiding X-radiation exposure.

In addition to improved lesion detection rates, 3D imaging can be valuable in improving carious treatment by minimizing the removal of tooth structure. Since the 3D OCT can pinpoint the lesion location in three dimensions, this can aid the selection of a less invasive and more precise treatment approach (Fig. 8). Better 3D guidance for caries management appears to reduce complications and decrease the procedure time. Moreover, 3D images can localize incipient demineralization and allow the dentist to perform the most conservative treatment possible.

Since 3D OCT can provide noninvasive image, the effect of remineralization therapy on enamel demineralization can be monitored to manage the lesion extent and depth three dimensionally. In this study, 3D OCT showed significantly higher sensitivity and specificity for the detection of enamel demineralization than the digital radiography. Several lines of evidence have shown that OCT measurements are smaller than histological measurements^21,22^; as a result, OCT is capable of imaging subclinical conditions and allows the early detection of dental diseases. Minimal intervention is crucial concept in modern dentistry for preserving dental hard tissue to ensure a longest tooth survival^5^. Because caries initiation and progression is a dynamic process involving alternating the balance of demineralization and remineralization, the selection of remineralization strategies should be based on an accurate diagnosis even for proximal caries of posterior teeth where visual inspection is difficult to achieve. Efforts to arrest and subsequently remineralize noncavitated lesions with prompt provision of preventive care are essential for minimize surgical intervention^3,8^. Recently, a micro-invasive treatment with low-viscosity resin infiltrant has clinically become one of the options for non-cavitated enamel caries^51^. Since resin infiltrant has proven to increase micro-hardness and reduce mineral loss after a demineralization challenge compared with untreated lesions^52^, it is considered to be efficacious in preventing further demineralization of proximal enamel caries. By contrast, treatment efficacy of resin infiltration appears influenced by the lesion depth and inhomogeneous for the progressed enamel caries^53,54^. A study of 3-year randomized clinical trial reported that the resin infiltrantion’s capacity to arrest noncavitated caries is reduced to 64% for lesions progressions in reaching outer dentin^55^. Consequently, accurate diagnosis of enamel caries regarding the depth and size appears crucial for the determination of less-invasive or micro-invasive approach. From this viewpoint, OCT appears to be a suitable method for the diagnosis of proximal enamel lesions and monitor the efficiency of non-invasive or micro-invasive therapy^8,53^.

However, OCT has some inherent limitations. Despite OCT being able to identify carious lesions up to the dentin level, it cannot clearly display the pulp chamber to determine the proximity of the lesion to the dental pulp^20^. Consequently, radiographic examination is still considered necessary for patients exhibiting symptoms of irreversible pulpitis. Moreover, nodulous topography of occlusal surface may disrupt the imaging depth of OCT for deep structure, when OCT viewing is performed from the occlusal aspect. Compared with our previous study on the detection of occlusal caries using 3D OCT image^44^, this study on proximal caries exhibited higher sensitivity for both cavitated enamel caries and dentin caries. However, no significant difference of specificity was found for all the diagnostic thresholds of caries level. Despite all the caries used in this study were natural caries on smooth tooth surface, it should be noted that many of the cases were located near or under the proximal contacts. It is probably because between the contact point and the top of the free gingival margin is the high risk for caries at smooth enamel surface^7,56,57^. While root caries at interproximal surface was not included in this study, different scanning protocol appears necessary for the imaging of root caries. For root caries, OCT image can be acquired from the cervical surface buccally or lingually if deems necessary^58,59^. Since OCT image has no risk for ionizing radiation, additional images with an altered position or scanning angle can be performed. 3D OCT can noninvasively provide cross-sectional views of internal tooth structures without unnecessary radiation exposure. Accordingly, it obviously becomes the safer diagnostic option that can be applied to the patients such as pregnant women, young children or where X-rays cannot be used such as aged care facilities.

The null hypothesis (H0) was rejected and alternative hypothesis (H1) was accepted in this study. 3D imaging of OCT could provide higher sensitivity and AUC for the diagnosis of proximal caries in posterior teeth than the intraoral digital radiography for the lesion level up to dentin caries. Moreover, 3D OCT showed higher diagnostic accuracy than the intraoral digital radiography. The modern imaging technology provides the opportunity to detect proximal caries on 3D images, thus overcoming the limitations of conventional 2D methods.

## Methods

### Study design

Ethical approval for this study design was obtained from the Institutional Review Board of Tokyo Medical and Dental University (approval number 578) in accordance with the guidelines Ethical Guidelines for Medical and Health Research involving Human Subjects. Extracted human third molars stored at 4 °C in water containing 0.02% sodium azide were used in this study. Written informed consent was obtained from the donors before the collection of teeth. The usage of the teeth was also reviewed and approved by the Institutional Review Board of Tokyo Medical and Dental University (approval number 725).

The proximal surfaces of the teeth were visually inspected with 2.5 × magnification loups and classified using ICDAS II. Both caries-free teeth (code 0) and teeth with caries (codes 1–5) were used for this study. From the teeth obtained, a total of 51 proximal surfaces (ICDAS code 0: 6 surfaces, code 1: 6 surfaces, code 2: 10 surfaces, code 3: 14 surfaces, code 4: 8 surfaces, code 5: 7 surfaces) of 36 molars were selected for investigation.

After the removal of calculus or debris from the surface, the teeth were mounted in silicone blocks (Exafine putty type, GC, Tokyo, Japan) at the same vertical level with the proximal surfaces in contact to simulate normal anatomical positions in the oral cavity. Since the proximal surfaces with or without carious lesions were in contact with those of neighboring teeth, visual assessment of the area of interest was impossible from the occlusal surface.

Digital photographs capturing the occluso-proximal surfaces of the mounted teeth were obtained under standardized conditions. After taking photographs, intraoral digital radiographs of the proximal surfaces were acquired, followed by 3D SS-OCT imaging of the areas of interest.

### Intraoral digital radiography

Digital radiographs of the proximal surfaces were captured with the X-ray tube positioned from the buccal surface at a distance of 40 cm replicating the position used in clinical practice. A digital intraoral sensor (Snapshot Instrumentarium, Kavo Dental, Biberach, Germany) was placed on the lingual aspect of the mounted teeth and then the X-ray unit (Dentnavi Hands XD35, Yoshida Dental, Tokyo, Japan) was operated at exposure settings of 60 kV, 7 mA, with an average exposure time of 0.6 s. Images were viewed using the associated image program software (ClinicalView ver.10.1, Instrumentarium, Kavo Dental) For each set of teeth, the proximal areas were imaged through a 1.0-cm thick water phantom in order to represent oral soft tissues.

### 3D OCT system

Following the acquisition of digital radiographic images, 3D OCT images were acquired by scanning the proximal areas from the occlusal surface of the teeth. The teeth were kept moist during the acquisition of OCT image so as to simulate intraoral condition. The OCT system (Yoshida Dental OCT, Yoshida Dental) used in this experiment was swept-source OCT (Fig. 1a), wherein the light source is a tunable laser that sweeps near-infrared wavelength light within millisecond scan delays at kilohertz rates to measure the magnitude and time delay of reflected light to construct a depth profile^45^. The wavelength ranged from 1240 to 1380 nm, with a central wavelength of 1310 nm and a 50-kHz sweep rate. This SS-OCT system is capable of acquiring complete tomographic images of a volume (10 mm long × 10 mm wide × 8 mm deep of optical pass depth) in 3.4 s.

The power of the sample beam was 18 mW, and the system has a sensitivity of 100 dB. The optical resolution of the 3D data set in air was less than 11 µm in depth and 40 µm in lateral and axial dimensions. The high-speed frequency-swept laser was projected from occluso-proximal aspect onto the proximal contact areas using a right-angle hand-held probe designed for the imaging of posterior teeth (Fig. 1b). A dental mirror with front surface reflection was attached to the tip of the handpiece which enabled the imaging of posterior teeth. Backscattered light from the object was coupled back to the system, digitized over a time scale, and analyzed by Fourier transformation to reveal the depth information of the sample. Cross-sectional image was generated by a ratter scanning of light achieved by galvanometer-based scanner. A sequence of cross-sectional images obtained by the raster scanning enabled the generation of 3D volumetric datasets, thereby yielding 3D images.

### Evaluation of proximal caries in posterior teeth

For image evaluation, an LCD monitor was used to display either radiographs or OCT images, with the digital photographs on a separate monitor. For the radiographs, the original image without enhancement of contrast or brightness was used. For OCT, 3D images of proximal contacts from the occlusal aspect, as well as the sequence of two-dimensional (2D) tomographic images extracted from the 3D dataset were dynamically displayed in video format using a custom-developed software (KakumaViewer, Yoshida Dental). The display settings such as brightness and contrast were kept the same for all images and examiners.

Thirteen dentists, each with over 5 years clinical experience in cariology and operative dentistry participated in this study as examiners. The examiners included 6 dentists experienced in OCT study and 7 first time viewers for OCT images. The number of examiners was determined to provide the sufficient sample size for further statistical analysis with α error probability for less than 0.05 and power of 1-β error probability for more than 0.95. In order to reach a consensus regarding the diagnostic criteria, the principal evaluator (YS) discussed OCT imaging with the 13 dentists in a 1-h session. After the discussion, the examiners were given 10 extracted teeth to examine and diagnose carious lesions using OCT for calibration amongst the examiners. These teeth were not included in the main study. Each examiner then scored the level of caries on the basis of the radiography and 3D OCT images separately. The following 4-point rank scale was used to score the level of caries progression.

Score 0: Sound tooth surface. No radiolucency at the surface was observed radiographically. In OCT, the obtained signal was the same level as that for the surrounding normal enamel.

Score 1: Superficial enamel demineralization without cavitation. The radiolucency of enamel was limited to the enamel thickness radiographically. In OCT, the signal intensity within the enamel thickness was enhanced with no enamel surface loss.

Score 2: Localized enamel breakdown due to caries. The continuity of the enamel surface was interrupted at the proximal surface. Caries was observed radiographically to have penetrated to the inner enamel without involving the dentin. The OCT signal was intensified but remained within the enamel thickness.

Score 3: Dentin caries. The caries was observed radiographically to have progressed into the dentin. An intensified OCT signal was obtained beyond the dentinoenamel junction (DEJ).

The diagnoses were then validated by direct observation of histologically sectioned proximal surfaces stained with a caries detecting dye. The teeth were sectioned and trimmed from the buccal surface along the mesio-distal plane parallel to the tooth axis using wet silicon carbide papers under running water, followed by further polishing with diamond paste down to 3 μm. The cross-sectioned polished surface was at the center of the lesion site, and was stained with a 0.5% acid red solution (Nishika’s Caries Check Red, Nippon Shika Yakuhin) for 10 s. The surfaces were then washed for 10 s and observed at a magnification of × 30. The same 4-point scale described above was used to determine the presence and extent of proximal caries as follows.

Score 0: Sound tooth surface.

Score 1: Superficial enamel demineralization without cavitation.

Score 2: Localized enamel breakdown due to caries. With no carious dentin stained by the 0.5% acid red solution.

Score 3: Dentin caries. Demineralization or structural changes in the dentin, which was stained by the 0.5% acid red solution.

### Statistical analysis

Statistical analyses were performed using a statistical software package (SPSS-2 for Windows, SPSS, Chicago, IL, USA). Indices of sensitivity and specificity for the detection of enamel demineralization (score 0 versus 1–3), enamel caries (score 0–1 versus 2–3), and dentin caries (score 0–2 versus 3) were calculated for the diagnostic results obtained from 3D OCT image and intraoral digital radiography for each examiner. Using these sensitivity and specificity values, a receiver operating characteristic (ROC) analysis was performed to calculate the area under the ROC curve (AUC). Diagnostic accuracy of 3D OCT image and intraoral digital radiography were also calculated by the agreement with histological findings using weighted kappa.

The results obtained by 13 examiners were statistically analyzed using Kolmogorov–Smirnov test for the data distributions (α = 0.05) and compared non-parametrically using Mann–Whitney U test (α = 0.05).

## Supplementary information

Supplementary Video 1.

Supplementary Video 2.

Supplementary Video 3.

Supplementary Video 4.

Supplementary Video 5.

Supplementary Video 6.
